# Association between constipation and childhood nocturnal enuresis in Taiwan: a population-based matched case-control study

**DOI:** 10.1186/s12887-020-1939-z

**Published:** 2020-01-28

**Authors:** Yu-Chao Hsiao, Jen-Hung Wang, Chia-Ling Chang, Chia-Jung Hsieh, Ming-Chun Chen

**Affiliations:** 1 Department of Pediatrics, Hualien Tzu Chi Hospital, Buddhist Tzu Chi Medical Foundation, Hualien, Taiwan; 2 School of Medicine, Tzu Chi University, No. 707, Section 3, Chung Yang Road, Hualien City, Hualien, Taiwan; 3 Department of Medical Research, Hualien Tzu Chi Hospital, Buddhist Tzu Chi Medical Foundation, Hualien, Taiwan; 4 Management Office for Health Data, Clinical Trial Research Center (CTC), China Medical University Hospital, Hongkong, China; 5 Department of Public Health, Tzu Chi University, Hualien, Taiwan

**Keywords:** Constipation, Enuresis, Laxative prescription, Nocturnal enuresis

## Abstract

**Background:**

The relationship between constipation and childhood nocturnal enuresis (NE) has been previously reported; however, this relationship remains controversial. The present study aimed to evaluate the association between constipation and childhood NE.

**Methods:**

Data from the Longitudinal Health Insurance Database 2000 (LHID 2000) of Taiwan National Health Insurance Research Database from 2000 to 2013 were collected. A total of 2286 children were enrolled in this study: a case group of 1143 children aged 5–18 years who were diagnosed with NE (NE group) and an age- and sex-matched control group of 1143 children without NE. Conditional logistic regression and odds ratio (OR) for NE were used to examine the association between constipation and childhood NE.

**Results:**

The prevalence of NE in the case group (NE group, aged 5–18 years) was 1.03% from 2000 to 2013. The NE group had a higher percentage of constipation in 1 year before the diagnosis of NE. After stratification for sex, both boys and girls with constipation had higher OR for NE. With stratification for age, children aged 5–12 and 7–12 years had a higher OR for NE.

**Conclusions:**

Constipation is associated with childhood NE in Taiwan, particularly in children aged 5–7 and 7–12 years.

## Background

Enuresis is synonymous with nocturnal incontinence or nocturnal enuresis (NE), and is defined as intermittent incontinence during sleep in children aged ≥5 years [1, 2]. According to disease onset and associated lower urinary tract symptoms, most cases of child NE are present as primary enuresis and monosymptomatic forms [1]. Enuresis occurs more frequently in boys than in girls [3–5], and epidemiology studies have reported that 5–12% of school-age children have NE [4–6]. Enuresis was believed to be characterized by the delayed maturation of voiding control [7]. Reduced bladder capacity, the nocturnal secretion of antidiuretic hormone (ADH) deficiency, and impaired sleep arousal function are three reported etiologies for enuresis [7–9]. Enuresis without treatment influences the physical health of children as well as their mental health and school performance [10].

Constipation, with a prevalence rate ranging from 4 to 37% as reported previously, is also a common problem in children [8]. Functional constipation is the most common etiology in the pediatric population [11]. Although constipation is not a life-threatening problem, constipation-related symptoms influenced the quality of life in children.

Among children with NE, the reported prevalence rate of constipation ranged from 7.06% in a Turkish study [12] to 69.8% in a study conducted at a tertiary pediatric voiding dysfunction clinic in the United States [2]. Chronic constipation may cause detrusor muscle instability, which may be associated with enuresis or urine incontinence [13]. Treatment for constipation improved enuresis, as reported in several studies [12, 13]. The relationship between constipation and enuresis has been previously reported [9, 10, 13]; however, the results were controversial because of conflicting findings [8]. The present study is, to the best of our knowledge, the first population-based, case-control study evaluating the association between constipation and enuresis using a nationwide database.

## Methods

### Data source

National Health Research Institute (NHRI) has 23 million enrollees, which is more than 99% of the population of Taiwan. NHRI released Taiwan’s National Health Insurance Research Database (NHIRD), which comprises abundant health and medical treatment information of insurants such as outpatient treatment, inpatient treatment, medication, and surgical operation information for each enrollee. Longitudinal Health Insurance Database 2000 (LHID 2000), which randomly selected one million subjects from NHIRD to explore the association between constipation and childhood NE, was used in this study. All diagnoses in the database were coded according to the International Classification of Disease, Ninth Revision, Clinical Modification (ICD-9-CM). The Ethics Review Board approved this study of China Medical University (CMUH-104-REC2–115). The Research Ethics Committee of China Medical University and Hospital in Taiwan approved this study (CMUH-104-REC2–115-R3).

### Study population

**Fig. 1:**
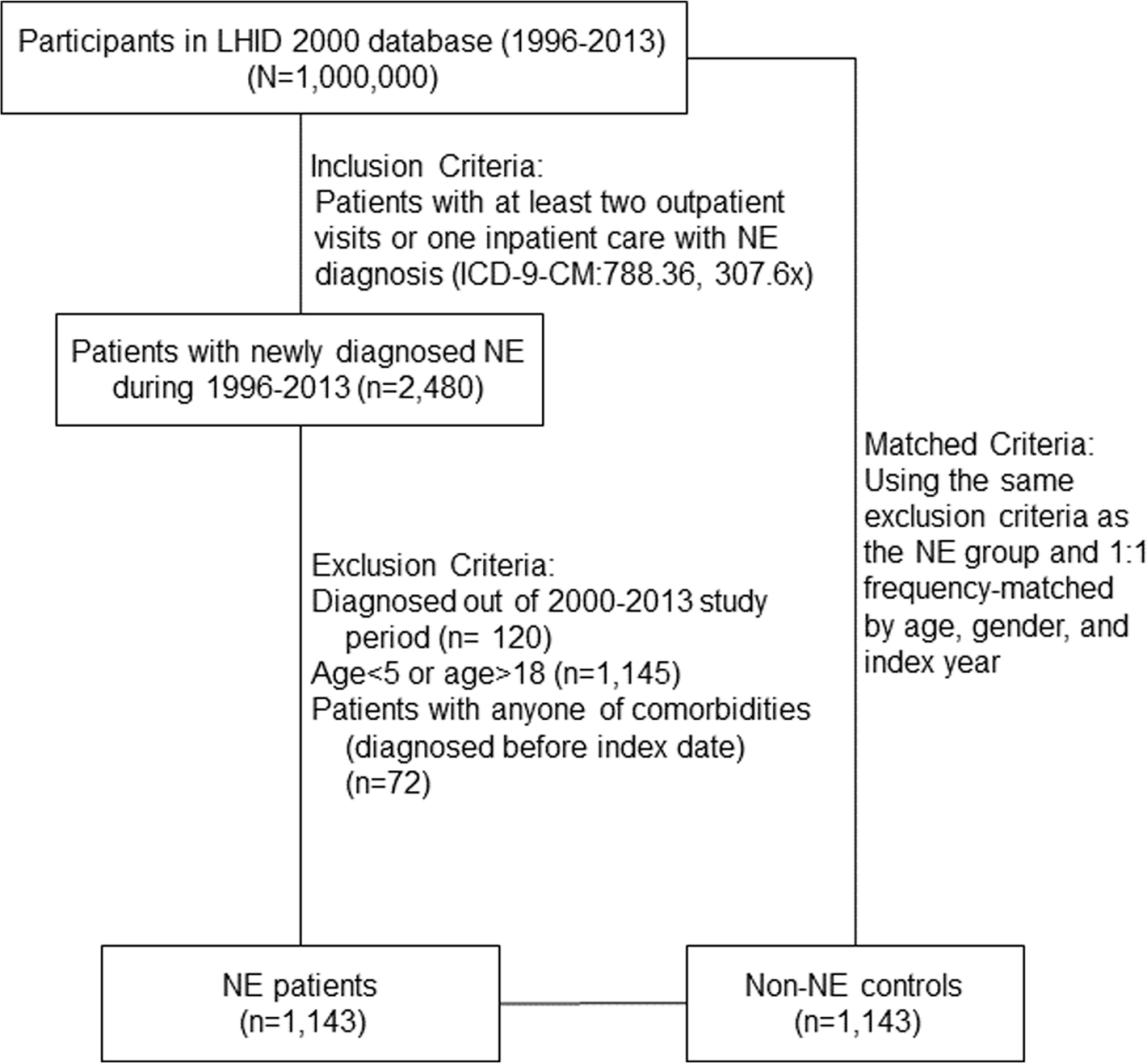
Flowchart of case and control participant recruitment.

The NE group enrolled children (aged 5–18 years) who made at least two outpatient visits or one inpatient visit for the diagnosis of NE (ICD-9-CM code 788.36, 307.6x) from January 2000 to December 2013. Subjects with comorbidities, including cerebral degeneration disorders (ICD-9-CM code 330, 331), neurogenic bladder (ICD-9-CM code 596.54), cauda equina syndrome with neurogenic bladder (ICD-9-CM code 344.61), mental retardation (ICD-9-CM code 317–319), congenital urinary tract anomaly (ICD-9-CM code 753), spinal bifida (ICD-9-CM code 741), or congenital anomalies of nervous system (ICD9-CM code 742), reported before the index date were excluded from the study. Subjects without NE were selected as the comparison group using the same exclusion criteria as the NE group and were 1:1 matched for sex, age, and index year. According to the definition provided in other studies that also used NHIRD, constipation was defined as a diagnosis of constipation (ICD-9-CM code 564.0) with laxative prescriptions (anatomical therapeutic chemical [ATC] classification system code A06A or A02AA02) within oneyear before the index date [14–17]. A total of 1143 NE patients and 1143 controls were enrolled in this study (Fig. 1).

### Statistical analysis

Distributions of age, sex, urbanization level, and exposure status, including constipation diagnosis and laxative prescriptionin the NE and comparison groups were presented as number and percentage and evaluated using the chi-square and t-tests. Odds ratio (OR) and 95% confidence interval (95% CI) were estimated using a conditional logistic regression model to evaluate the association of constipation diagnosis and laxative prescriptions with NE. Analyses of stratification by sex and age were performed to examine the association of constipation/laxative prescriptions with NE among a specific population. All statistical analyses were performed using the STATA statistical software (StataCorp. 2015. Stata Statistical Software: Release 14. College Station, TX: StataCorp LP). The two-tailed test was used to determine statistical significance (*p* < 0.05).

## Results

Table 1 presents the demographic characteristics and distribution of sex, age, urbanization, and exposure status among patients. A total of 1143 patients were included in the case and control groups each, and boys accounted for 59% of the population in each group. The mean age of the study population was 8.8 years. In the comparison between the NE and comparison group, a significantly different distribution of exposure status, including times of diagnosis of constipation, times of laxative prescription, and constipation diagnosis with laxative prescriptions, was noted. The prevalence of constipation diagnosis and the laxative prescription was higher in the NE group than in the comparison group (7.1% vs. 3.9 and 14.1% vs. 8.5%). Additionally, the NE group had a higher prevalence of constipation diagnosis with laxative prescriptions in 1 year before the diagnosis of NE (5.3% vs. 3.0%) (Table 1) than the comparison group. Most children in both the NE and non-NE groups only had one diagnosis of constipation and 1–2 times of laxative prescriptions within one year.

**Table 1:** Demographic characteristics and association between constipation and childhood NE

	childhood NE		
	No (*n* = 1143)	Yes (*n* = 1143)	
	*N* (%)	*N* (%)	*p* value^a^
Gender^a^			> 0.99
Female	469 (41.0)	469 (41.0)	
Male	674 (59.0)	674 (59.0)	
Age^a^			0.839
5–7	325 (28.4)	313 (27.4)	
7–12	697 (61.0)	710 (62.1)	
12–18	121 (10.6)	120 (10.5)	
mean (SD)^b^	8.8 (0.08)	8.8 (0.07)	0.943^b^
Urbanization level			0.326
1 (highest)	325 (28.5)	297 (26.0)	
2	343 (30.0)	345 (30.2)	
3	220 (19.3)	231 (20.2)	
4	142 (12.4)	164 (14.4)	
5	16 (1.4)	22 (1.9)	
6	42 (3.7)	45 (3.9)	
7 (lowest)	54 (4.7)	38 (3.3)	
Constipation diagnosis in one year^a^			0.007
No	1098 (96.1)	1062 (92.9)	
Yes (times)	45 (3.9)	81 (7.1)	
1	31 (2.7)	56 (4.9)	
2	11 (1.0)	15 (1.3)	
≥ 3	3 (0.3)	10 (0.9)	
Laxative prescriptions in one year^a^			< 0.0001
No	1046 (91.5)	982 (85.9)	
Yes (times)	97 (8.5)	161 (14.1)	
1–2	78 (6.8)	129 (11.3)	
3–5	17 (1.5)	23 (2.0)	
≥ 6	2 (0.2)	9 (0.8)	
Constipation diagnosis and Laxative prescriptions^a^			0.006
No	1109 (97.0)	1083 (94.8)	
Yes	34 (3.0)	60 (5.3)	

^a^Tested by chi-square test

^b^Tested by T test

Table 2 presents the ORs of estimated NE odds based on constipation according to conditional logistic regression. NE subjects had a significantly higher OR (OR: 1.79, 95% CI: 1.17–2.74, *p = 0.008*) for constipation than non-NE controls. The prevalence of NE in children aged 5–18 years was 1.03% during 2000–2013 (data not shown).

**Table 2:** Conditional logistic regression model for evaluating the association between constipation diagnosis and laxative prescriptions and childhood NE

	Univariate	
	OR (95% CI)	*p* value
Constipation diagnosis and Laxative prescriptions		
No	reference	
Yes	1.79 (1.17–2.74)	0.008

The association between constipation and NE was evaluated using stratification analysis, wherein data were stratified by sex and age (Table 3). Girls with constipation had a significantly higher OR for NE (OR: 2.34, 95% CI: 1.58–3.48, *p < 0.001)* than girls without constipation. Similarly, boys with constipation had a significantly higher OR for NE (OR: 1.52, 95% CI: 1.07–2.16, *p = 0.019*) than boys without constipation. In subgroup analyses for children in different age groups, constipation was significantly associated with NE in children aged 5–7 and 7–12 years (OR: 1.67, 95% CI: 1.03–2.69, *p = 0.036*;OR: 1.81, 95% CI: 1.27–2.58, *p = 0.001,* respectively) but not in children aged 12–18 years (OR: 1.17, 95% CI: 0.39–3.47, *p* = 0.782).

**Table 3:** Associations between constipation diagnosis and laxative prescriptions and childhood NE, stratified by sex and age

	Constipation diagnosis and Laxative prescriptions	
	OR (95% CI)	*p* value
Gender		
Female	2.34 (1.58–3.48)	< 0.001
Male	1.52 (1.07–2.16)	0.019
Age		
5–7	1.67 (1.03–2.69)	0.036
7–12	1.81 (1.27–2.58)	0.001
12–18	1.17 (0.39–3.47)	0.782

## Discussion

In the present study, a higher constipation rate was noted within one year before the diagnosis of NE in the NE group compared with the non-NE group. Both boys and girls with constipation had a significantly higher risk for developing NE, and children with constipation aged 5–7 and 7–12 years had a relatively and significantly higher OR for NE.

Determining the prevalence of NE is challenging. Previous studies have revealed that the prevalence rate of NE was approximately 5–12% in Taiwan and other countries and that this rate decreased gradually with increased age [3, 5, 18]. Although the prevalence rate of NE in the present study (1.03%; data not shown) based on the NHIRD in Taiwan is markedly lower than that reported in the studies mentioned above, it is the real prevalence rate that pediatricians encounter in everyday practice. The analyses of different databases may result in different prevalence rates for NE, and some studies have estimated the prevalence rate of NE using questionnaires administered to parents who may not know the definition of NE [8]. Some parents did not want medical intervention for their children because they believed that NE was a natural developmental condition and can be spontaneously resolved with age [8, 19]. For the above reasons, education and early intervention for NE are essential for ensuring the well-being of affected children and their families.

Previous epidemiology studies have revealed highly variable prevalence rates of pediatric constipation worldwide (0.7–69.8%), depending on the different geographic area [2, 8, 10, 20], inconsistent awareness of constipation [8, 10, 19], or the use of a different definition for diagnosis [2]. Although a relatively better definition for constipation is that stated by Rome Criteria, different definitions of constipation and different indications of treatment are still being used. The definition in the present study using “the diagnosis of constipation and the use of laxative drugs”can be more practical for a clinical condition for medical intervention of constipation. The lower rate of constipation may be underreported considering that individuals may not seek advice out of embarrassment. However, previous studies have also demonstrated that only a small number of children with constipation accepted medical treatment, which is in line with the findings of the present study [19, 20].

Our study revealed a significant association between constipation and NE in a pediatric population. The prevalence rate of NE gradually decreases each year [5]. Although NE is less common among adolescents, the etiology of NE in adolescents was more complex than that in younger children. A nationwide epidemiological study of NE in Korea reported that bladder function, sleep disturbance, and family history of NE were risk factors for adolescent NE [21]. Therefore, the influence of constipation among adolescents with NE may be less prominent. Besides, our data also revealed that both boys and girls with constipation had a significant association with NE, and a higher OR was noted in girls than in boys (2.34 vs. 1.52). This phenomenon was rarely reported in previous studies, and the probable reason for this is unclear. Further studies are warranted to determine if the association between constipation and NE is more prominent in girls than in boys.

Previous studies have revealed that children with constipation had higher urinary incontinence rates than children without constipation [13]. Although our data showed a low constipation rate in both NE and non-NE groups, children with constipation had a significantly higher OR (1.79) for NE than children without constipation. Recent studies revealed the severity of NE was significantly positively associated with the presence of constipation [22, 23]. With the successful treatment of constipation, nighttime urinary incontinence disappeared in 63% of the patients [13]. Moreover, constipation was significantly associated with the effectiveness of treatment with desmopressin for patients with NE [23]. In brief, constipation treatment played an important, if not a major role, in the management of NE in children with chronic constipation [5, 13].

The mechanism by which constipation affects the pathogenesis of NE is possibly multifactorial. Anatomically, the bladder and rectum are both located in the pelvic area, which is relatively small in children [10]. Chronic constipation may cause a distended rectum, which may then compress the bladder and decrease bladder capacity [13, 24], thereby increasing detrusor instability. Urodynamic studies have demonstrated detrusor instability in children with NE and constipation [25]. Detrusor instability due to nocturnal colonic movement may be more obvious at night [10]. Most children with chronic constipation have increased residual urine after voiding, as noted by ultrasound, and treatment of constipation can decrease the amount of postvoiding residual urine [26]. Taken together, constipation-induced distended rectum and direct compression of the bladder may aggravate the severity of NE and impair the effectiveness of treatment for NE. However, some children still had NE after the management of distended rectum [27]; as a result, other studies have reported that the relationship of neuromuscular function between voiding and bowel defecation is also associated with the occurrence of constipation and NE [24, 27]. Autonomic nerves, namely sympathetic and parasympathetic nerves, control urethral and anal sphincter functions. Autonomic incoordination may induce chronic constipation and lower urinary tract symptoms, including NE [24, 27]. Both urethral and anal sphincters innervated by the autonomic nerve share the associated pelvic floor muscle. Constipation was considered to be associated with the inappropriate contraction of the pelvic floor muscle [11], which would accompany the nonrelaxation of the urethral sphincter. Incoordination between urethral sphincter relaxation and detrusor contraction would subsequently cause NE [13, 24, 25, 27].

The present study revealed a significant association between constipation and NE in children, which may shed some light on the clinical approach and treatment for children with NE. Evaluation of constipation is needed before and during NE management [5, 27, 28]. We should inquire about constipation-associated symptoms instead of only discussing “constipation.” Bristol Stool Form Scale is a good tool to help obtain better descriptions of stool from patients. A bowel and voiding diary could offer better information about bowel health and NE condition [27]. Frequent evaluation of bowel health is important because pharmacological treatment failure may be associated with constipation in patients with NE. Alarm therapy and desmopressin are useful in the management of child NE. However, constipation in children with NE may decrease the success rate of these managements of NE [20, 23]. Oxybutynin, an anticholinergic agent, which increases functional bladder capacity, may be used for children with NE in case of poor response to alarm therapy and desmopressin. Unfortunately, some children have constipation as a side effect of oxybutynin [2, 5], which may aggravate the severity and occurrence of NE. As a result, the patient’s bowel health must be evaluated before and during the treatment and must be re-evaluated if the patient responds poorly to various NE treatments.

### Limitation of studies

Our study was a nationwide population-based study with a large sample size, and it revealed the possible association between constipation and child NE. Nevertheless, there are some limitations to our research. First, the definition of *constipation* may affect the prevalence and results of the study. Presently, the most accepted definition for constipation is the Rome IV criteria; however, detailed information about constipation-related history including in the Rome criteria are not available in our database. Besides laboratory and imaging data, information on the patient’s exposure factors, such as diet, fluid intake, exercise, and the use of complementary medications, is not available in our database. This information may pose some bias in our study. We defined constipation using both the ICD-9 diagnostic code and the ATC prescription code, as in previous studies, to minimize bias [14–17]. Second, children with constipation and/or NE who did not receive the medical intervention or not use prescription medicine would not be recorded in the NHIRD, which may underestimate the true prevalence of both diseases. However, it is the real prevalence that pediatricians encounter in everyday practice as we have mentioned earlier. Third, previous studies have revealed that allergic diseases, sleep disorders, and psychotic disorders may be associated with NE [29, 30]. Because these associations were inconclusive, we did not consider these factors in our study. Due to the abovementioned limitations, further studies are required for causality between constipation and child NE.

## Conclusion

This population-based, matched case-control study revealed a possible association between constipation and NE in children. Children with NE, particularly those aged between 5 and 12 years, had a higher constipation rate compared with children without NE. More clinical trials or prospective studies are required for the evaluation of the causal relationship between constipation and NE.

## Data Availability

All data generated or analyzed during this study are included in this published article.

## Abbreviations

ATC: Anatomical therapeutic chemical
NE: Nocturnal enuresis
NHIRD: National Health Insurance Research Database
NHRI: National Health Research Institute
